# Association between non-high-density lipoprotein cholesterol to high-density lipoprotein cholesterol ratio and osteoporotic fracture

**DOI:** 10.3389/fmed.2026.1754715

**Published:** 2026-03-31

**Authors:** Jie Deng, Mengmeng Shang, Junhao Jin, Suhong Wei

**Affiliations:** 1 The First School of Clinical Medicine, Lanzhou University, Lanzhou, China; 2 Department of Endocrinology, Gansu Provincial Hospital, Lanzhou, China; 3 The First Clinical Medical College, Gansu University of Traditional Chinese Medicine, Lanzhou, China; 4 The Third Hospital of Lanzhou University, Lanzhou, China

**Keywords:** bone metabolism, lipid metabolism, non-high-density lipoprotein cholesterol to high-density lipoprotein cholesterol ratio, osteoporotic fracture, retrospective cross-sectional study

## Abstract

**Background:**

The non-high-density lipoprotein cholesterol/high-density lipoprotein cholesterol ratio (NHHR) has emerged as a valuable lipid marker associated with various cardiometabolic diseases. Although evidence links lipid metabolism to skeletal health, the relationship between NHHR and osteoporotic fractures remains unexplored. This study aims to assess the association between NHHR and the risk of osteoporotic fractures.

**Method:**

This retrospective cross-sectional study included 580 patients from the Department of Endocrinology at Gansu Provincial People's Hospital between January 2020 and December 2024. The association between NHHR and osteoporotic fracture was assessed using multivariate logistic regression with covariate adjustment. RCS analysis explored non-linear relationships, and comprehensive subgroup analyses validated the findings' consistency.

**Results:**

In fully adjusted multivariate logistic regression models, NHHR demonstrated a significant inverse association with osteoporotic fracture (OR = 0.55, 95% CI: 0.45–0.66, *p* < 0.001), with each one-unit increment corresponding to a 45% reduction in fracture odds. Categorical analysis by NHHR quartiles confirmed a dose-response relationship, with participants in the highest quartile exhibiting 80% lower odds of fracture compared to the reference lowest quartile (OR = 0.20, 95% CI: 0.11–0.36, *p* < 0.001). Restricted cubic spline regression revealed a significant non-linear relationship (*p* for non-linearity < 0.001) with an inflection point identified at NHHR = 3.29. In stratified analyses, the inverse association between NHHR and osteoporotic fracture persisted across all demographic subgroups.

**Conclusions:**

Our study identified a significant non-linear association between NHHR and osteoporotic fracture risk. Due to its retrospective nature, further prospective investigations are needed to confirm these [r]results.

## Introduction

Osteoporosis is a systemic skeletal disorder marked by decreased bone mass and deterioration of bone microarchitecture, ultimately resulting in reduced bone strength and increased susceptibility to fractures (1, 2). As the global population ages rapidly (3), osteoporotic fractures have emerged as a significant public health challenge, affecting approximately nine million individuals aged 50 and above each year (4, 5). These fractures place an increasing economic and resource burden on healthcare systems worldwide (6, 7). Notably, in China, cases are projected to reach 5.99 million by 2050, with associated medical costs estimated at US$25.43 billion (8). Consequently, the early identification of risk factors and the discovery of reliable biomarkers are urgently needed for effective risk stratification and intervention.

However, studies examining the association between individual lipid parameters and osteoporotic fracture risk have produced inconsistent results (9, 10), highlighting the limitations of traditional lipid indicators in assessing the impact of lipid metabolism on bone health (11, 12). NHHR is a novel composite indicator that integrates both pro-atherogenic and anti-atherogenic lipid components, thereby providing a more comprehensive reflection of systemic lipid metabolic status (13). NHHR has been shown to be closely linked to various metabolic diseases, such as diabetic nephropathy and non-alcoholic fatty liver disease (14, 15). Nevertheless, its relationship with osteoporotic fractures has not yet been systematically investigated. Therefore, this study aims to systematically assess the association between NHHR and the risk of osteoporotic fractures, in order to explore its potential utility as a novel biomarker for early detection and targeted prevention.

## Materials and methods

### Study design and population

**Figure 1:**
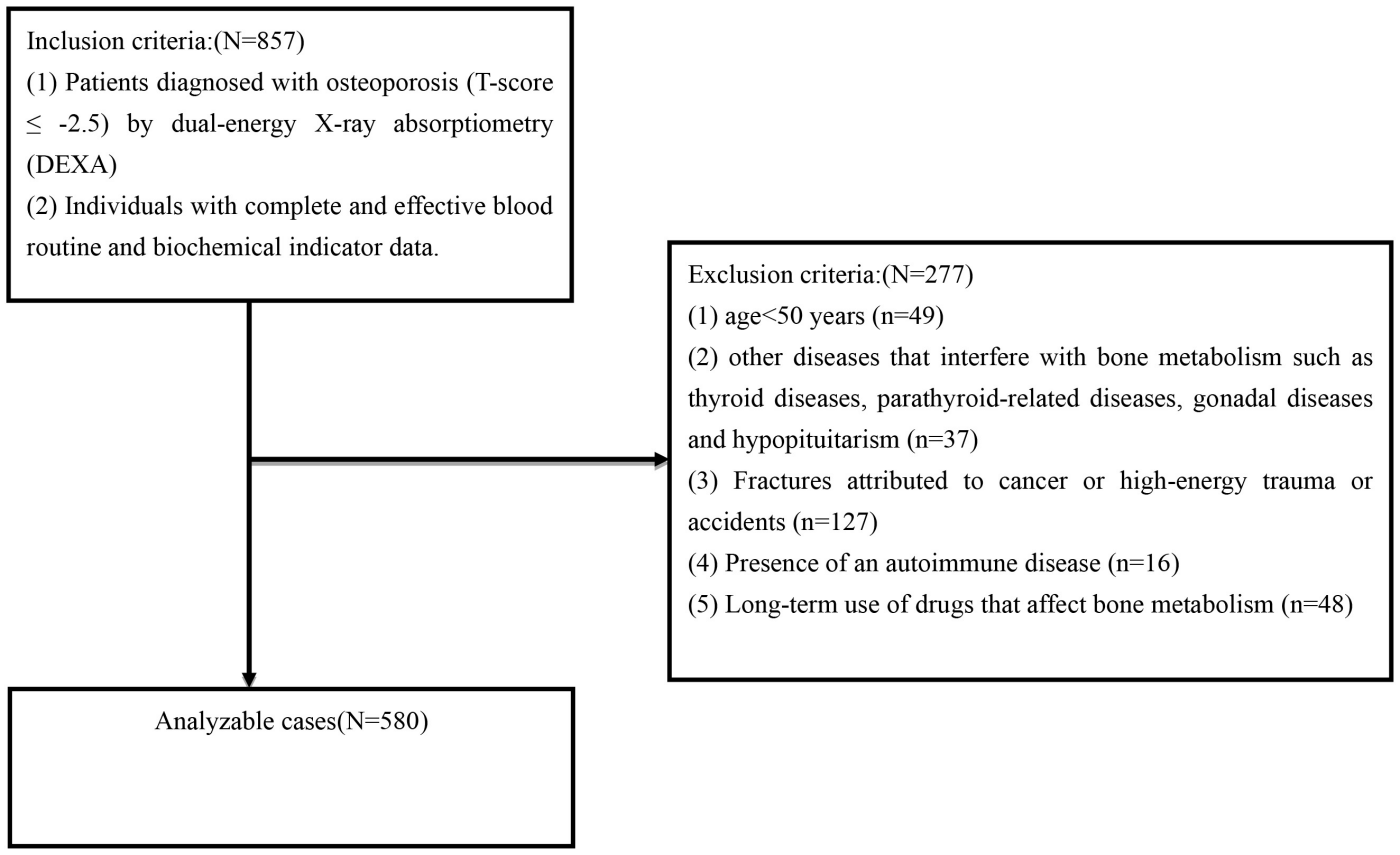
Flowchart of participant selection.

This retrospective cross-sectional study included patients from the Department of Endocrinology at Gansu Provincial People's Hospital between January 2020 and December 2024. The study protocol was approved by the Institutional Ethics Committee of Gansu Provincial People's Hospital (Approval No. 2025-011) and conducted in accordance with the Declaration of Helsinki. Eligible participants met the following inclusion criteria: (1) diagnosis of osteoporosis, defined as a *T*-score ≤ -2.5 by dual-energy X-ray absorptiometry (DXA); and (2) availability of complete and reliable hematological and biochemical profiles. The exclusion criteria were: (1) age below 50 years; (2) comorbid conditions affecting bone metabolism, including thyroid disorders, parathyroid dysfunction, gonadal diseases, or hypopituitarism;(3) fractures attributable to malignancy, high-energy trauma, or accidental injury; (4) autoimmune diseases such as systemic lupus erythematosus; and (5) chronic use of medications known to affect bone metabolism. Ultimately, 580 patients were included in the final analysis (Figure 1).

### Sample size calculation

The minimum required sample size was calculated using the standard formula for cross-sectional studies.


$$\begin{array}{l} N=\frac{z_{1-\alpha /2}^{2}\;P\;\left(1-P\right)}{d^{2}} \end{array}$$ (1)

where $z_{1-\alpha /2}^{2}$ represents the standard normal deviate corresponding to the two-sided significance level α, *p* is the expected prevalence, and *d* denotes the desired precision. We set α = 0.05 (i.e., $z_{1-\alpha /2}^{2}=$ 1.96) and *d* = 0.05. Based on assumed prevalence of *p* = 0.247, the minimum required sample size was 286.

### Assessment of NHHR

The NHHR was calculated as follows: NHHR = (total cholesterol [TC] – high-density lipoprotein cholesterol [HDL-C])/HDL-C. TC and HDL-C were measured from fasting blood samples using standardized enzymatic colorimetric assays and chemiluminescent immunoassays.

### Assessment of incident osteoporotic fracture

All patients in this study had confirmed osteoporosis, defined as a *T*-score ≤ -2.5 by dual-energy X-ray absorptiometry (DXA). To ensure diagnostic specificity, individuals with other metabolic bone disorders (including osteomalacia, Paget's disease of bone, osteogenesis imperfecta, and other secondary metabolic bone diseases) were excluded. Osteoporotic fractures were defined as fragility fractures resulting from low-energy trauma, such as a fall from standing height or less (16). For outcome assessment, fractures at any skeletal site were included, except those involving the toes, digits, or facial bones, to ensure comprehensive capture of clinically relevant events while excluding sites less likely to reflect osteoporosis-related risk.

### Covariate analyses

Potential confounders included demographic factors [sex, age, body mass index (BMI)], comorbidities [hypertension and type 2 diabetes mellitus (T2DM)], bone turnover markers [osteocalcin (OC), β-C-terminal telopeptide of type I collagen (β-CTX), and parathyroid hormone (PTH)], and biochemical indicators [aspartate aminotransferase (AST), alkaline phosphatase (ALP), calcium (Ca), potassium (K), sodium (Na), phosphorus (P), and hemoglobin (Hb)]. BMI was calculated as weight (kg) divided by height squared (m^2^).

Fasting blood samples were obtained after an overnight fast of at least 8 h. Serum biochemical parameters (AST, ALP, Ca, K, Na, P) were analyzed using an automated biochemical analyzer (Abbott C-1600). Bone turnover markers [OC, β-CTX, PTH) and 25-hydroxyvitamin D (25(OH)D] were measured via chemiluminescence immunoassay (Abbott ISR-2000). Hemoglobin was assessed using a Sysmex XN-9000 hematology analyzer. Bone mineral density (BMD) measurements at the lumbar spine, femoral neck, and total hip were performed by dual-energy X-ray absorptiometry (DXA).

### Statistical analysis

Statistical analyses were performed according to data distribution characteristics. Continuous variables that followed a normal distribution were compared using the independent samples t-test and presented as mean ± standard deviation (SD). Variables with non-normal distribution were compared using the Mann–Whitney *U*-test and presented as median (interquartile range, IQR). Categorical variables were reported as frequencies and percentages, and univariate analysis was performed using Pearson's chi-square test or Fisher's exact test. Logistic regression analysis was employed to explore the relationship between NHHR and osteoporotic fracture after adjusting for the covariates mentioned previously. Covariates were selected a priori based on clinical relevance and previous evidence linking lipid metabolism and osteoporotic fracture risk. Covariate selection and model building followed standard methodological recommendations. Following the guidelines of the Strengthening the Reporting of Observational Studies in Epidemiology (STROBE) statement, our analytical results were presented in three distinct models: an unadjusted model, a partially adjusted model accounting for basic confounders, and a fully adjusted model incorporating all potential covariates. The presence of multicollinearity was tested using the variance inflation factor (VIF) method, where a VIF ≥10 indicates multicollinearity. Odds ratios (ORs) were selected as the primary measure given the cross-sectional design's focus on fracture prevalence rather than incidence. The OR and 95% confidence interval (CI) were calculated. Subgroup analysis were performed to assess effect modifications by predefined covariates (age, sex, BMI, hypertension, and diabetes) on the NHHR-fracture association, with interaction terms tested in multivariate models with a *p*-value < 0.05 indicating a significant interaction effect. Statistical analyses were performed using R software (version 4.2.3). A *p*-value of < 0.05 was considered indicative of statistical significance.

## Results

### Baseline characteristics

The characteristics of the participants are shown in the Table 1. The final study population comprised 580 participants who satisfied the eligibility criteria, with an average age of 65.13 ± 8.78 years. The majority of participants were female. Among these participants, 241 (41.6%) were diagnosed with osteoporotic fractures, while 339 (58.4%) had no fractures. Patients with fractures were older and had significantly lower BMD at the lumbar spine and hip. Compared with the non-fracture group, the fracture group exhibited lower NHHR levels, higher HDL. No significant differences in OC, β-CTX, or 25(OH)D were observed between the two groups.

**Table 1:** Baseline characteristics of the study population.

Variables	Total (*n* = 580)	Non-fracture group (*n* = 339)	Fracture group (*n* = 241)	*P*
				
Age, years	65.13 ± 8.78	64.28 ± 9.00	66.33 ± 8.33	0.006^a^
BMI, kg/m^2^	22.53 ± 2.78	22.61 ± 2.73	22.41 ± 2.85	0.396^a^
Oc, ng/ml	17.49 ± 8.32	17.51 ± 8.12	17.47 ± 8.60	0.949^a^
β-CTX, ng/ml	0.46 ± 0.28	0.47 ± 0.27	0.45 ± 0.29	0.451^a^
25(OH)D, ng/ml	18.13 ± 8.58	17.72 ± 8.60	18.70 ± 8.53	0.175^a^
PTH, pg/ml	55.30 ± 24.00	54.57 ± 24.62	56.34 ± 23.12	0.383^a^
Ca, mmol/L	2.30 ± 0.11	2.30 ± 0.11	2.31 ± 0.11	0.746^a^
P, mmol/L	1.15 ± 0.17	1.14 ± 0.17	1.15 ± 0.16	0.788^a^
L1BMD, g/cm^2^	0.77 ± 0.15	0.78 ± 0.13	0.76 ± 0.16	0.030^a^
L2BMD, g/cm^2^	0.81 ± 0.13	0.83 ± 0.12	0.78 ± 0.14	< 0.001^a^
L3BMD, g/cm^2^	0.87 ± 0.13	0.91 ± 0.13	0.83 ± 0.13	< 0.001^a^
L4BMD, g/cm^2^	0.90 ± 0.16	0.93 ± 0.16	0.86 ± 0.15	< 0.001^a^
L1-4BMD, g/cm^2^	0.83 ± 0.11	0.85 ± 0.10	0.80 ± 0.11	< 0.001^a^
FN BMD, g/cm^2^	0.71 ± 0.09	0.73 ± 0.08	0.69 ± 0.10	< 0.001^a^
Hip BMD, g/cm^2^	0.78 ± 0.11	0.80 ± 0.09	0.74 ± 0.11	< 0.001^a^
TP, g/L	71.61 ± 7.33	71.18 ± 7.58	72.20 ± 6.9	0.098^a^
AST, U/L	22.25 ± 8.62	21.75 ± 8.88	22.94 ± 8.21	0.101^a^
ALP, U/L	85.81 ± 29.57	83.72 ± 29.57	88.74 ± 29.39	0.044^a^
K, mmol/L	4.01 ± 0.35	4.04 ± 0.37	3.98 ± 0.31	0.052^a^
Na, mmol/L	140.29 ± 2.36	140.14 ± 2.35	140.50 ± 2.36	0.072^a^
HDL, mmol/L	1.13 ± 0.30	1.09 ± 0.29	1.17 ± 0.31	< 0.001^a^
LDL, mmol/L	2.40 ± 0.78	2.43 ± 0.77	2.36 ± 0.80	0.299
WBC, 10^9^/L	6.08 ± 2.03	6.11 ± 1.83	6.05 ± 2.30	0.725^a^
Hb, g/L	139.99 ± 13.04	140.79 ± 12.75	138.85 ± 13.37	0.077^a^
Sex, *n* (%)				
Male	94 (16.21)	70 (20.65)	24 (9.96)	< 0.001^a^
Female	486 (83.79)	269 (79.35)	217 (90.04)	
Hypertension, *n* (%)				
No	395 (68.10)	234 (69.03)	161 (66.80)	0.572^b^
Yes	185 (31.90)	105 (30.97)	80 (33.20)	
T2DM, *n* (%)				
No	262 (45.17)	134 (39.53)	128 (53.11)	0.001^b^
Yes	318 (54.83)	205 (60.47)	113 (46.89)	
NHHR	3.15 ± 1.22	3.42 ± 1.36	2.78 ± 0.87	< 0.001^a^

BMI, body mass index; OC, osteocalcin; β-C-terminal telopeptide of type I collagen; 25(OH)D, 25-hydroxyvitamin D; PTH, parathyroid hormone; Ca, calcium; P, phosphorus; L1BMD, L1 vertebral body bone mineral density; L2BMD, L2 vertebral body bone mineral density; L3BMD, L3 vertebral body bone mineral density; L4BMD, L4 vertebral body bone mineral density; L1-4BMD, lumbar spine (L1 to L4) bone mineral density; FN BMD, Femoral neck bone mineral density; TP, total protein; AST, aspartate aminotransferase; ALP, alkaline phosphatase; K, potassium; Na, sodium; HDL, high-density lipoprotein; LDL, low-density lipoprotein; WBC, white blood cell count; Hb, hemoglobin; T2DM, type 2 diabetes mellitus; NHHR, non-high-density lipoprotein cholesterol to high-density lipoprotein cholesterol ratio.

^a^Independent samples t-test.

^b^Chi-square test.

### Association between the NHHR and osteoporotic fracture

The association between NHHR and the prevalence of osteoporotic fracture was evaluated using logistic regression models, as presented in Table 2. Multicollinearity among covariates was assessed by variance inflation factor (VIF), with all VIF values below five, suggesting no significant multicollinearity. Three hierarchical models were constructed to investigate this relationship. In the unadjusted model (Model 1), higher NHHR was significantly associated with lower odds of osteoporotic fracture (OR = 0.58, 95% CI: 0.49–0.69, *p* < 0.001). This inverse association persisted after adjustment for age, sex, hypertension, and diabetes in Model 2 (OR = 0.59, 95% CI: 0.49–0.71, *p* < 0.001). In the fully adjusted Model 3—which further controlled for OC, CTX, PTH, P, L1–L4 BMD, femoral neck BMD, AST, ALP, K, Na, Ca and Hb—each unit increase in NHHR was independently associated with a 45% reduction in the odds of osteoporotic fracture (OR = 0.55, 95% CI: 0.45–0.66, *p* < 0.001). When NHHR was analyzed as a categorical variable using quartiles, participants in the highest quartile (Q4) exhibited a significantly lower risk of osteoporotic fracture compared to those in the lowest quartile (Q1) in the fully adjusted model (OR = 0.20, 95% CI: 0.11–0.36, *p* < 0.001). A significant dose–response trend was observed, with the risk of osteoporotic fracture decreasing progressively across increasing NHHR quartiles (*p* for trend < 0.001). Our results indicate a significant inverse association between NHHR and osteoporotic fracture prevalence, independent of measured confounders.

**Table 2:** The relationship between NHHR and osteoporotic fracture.

Exposure	Model 1		Model 2		Model 3	
	**OR (95% CI)**	***p***	**OR (95% CI)**	***p***	**OR (95%CI)**	***p***
Continuous models						
NHHR	0.58 (0.49 ~ 0.69)	< 0.001	0.59 (0.49 ~ 0.71)	< 0.001	0.55 (0.45 ~ 0.66)	< 0.001
Categorical models						
Q1	[Reference]		[Reference]		[Reference]	
Q2	0.89 (0.55 ~ 1.43)	0.627	0.83 (0.51 ~ 1.35)	0.455	0.74 (0.44 ~ 1.24)	0.258
Q3	0.32 (0.20 ~ 0.52)	< 0.001	0.30 (0.18 ~ 0.50)	< 0.001	0.25 (0.14 ~ 0.43)	< 0.001
Q4	0.23 (0.13 ~ 0.39)	< 0.001	0.25 (0.14 ~ 0.43)	< 0.001	0.20 (0.11 ~ 0.36)	< 0.001
*P* for trend	< 0.001		< 0.001		< 0.001	

OR: Odds Ratio, CI: Confidence Interval; NHHR: non-high-density lipoprotein cholesterol to high-density lipoprotein cholesterol ratio. Model1: No covariates adjusted; Model2: Adjust for age, sex, BMI, hypertension, diabetes; Model3: Adjust for age, sex, BMI, hypertension, diabetes, OC, CTX, PTH, P, L1-L4BMD, Femoral neck BMD, AST, ALP, K, Na, Ca, Hb.

### Nonlinear relationship and saturation effect analysis between NHHR and osteoporotic fracture

**Figure 2:**
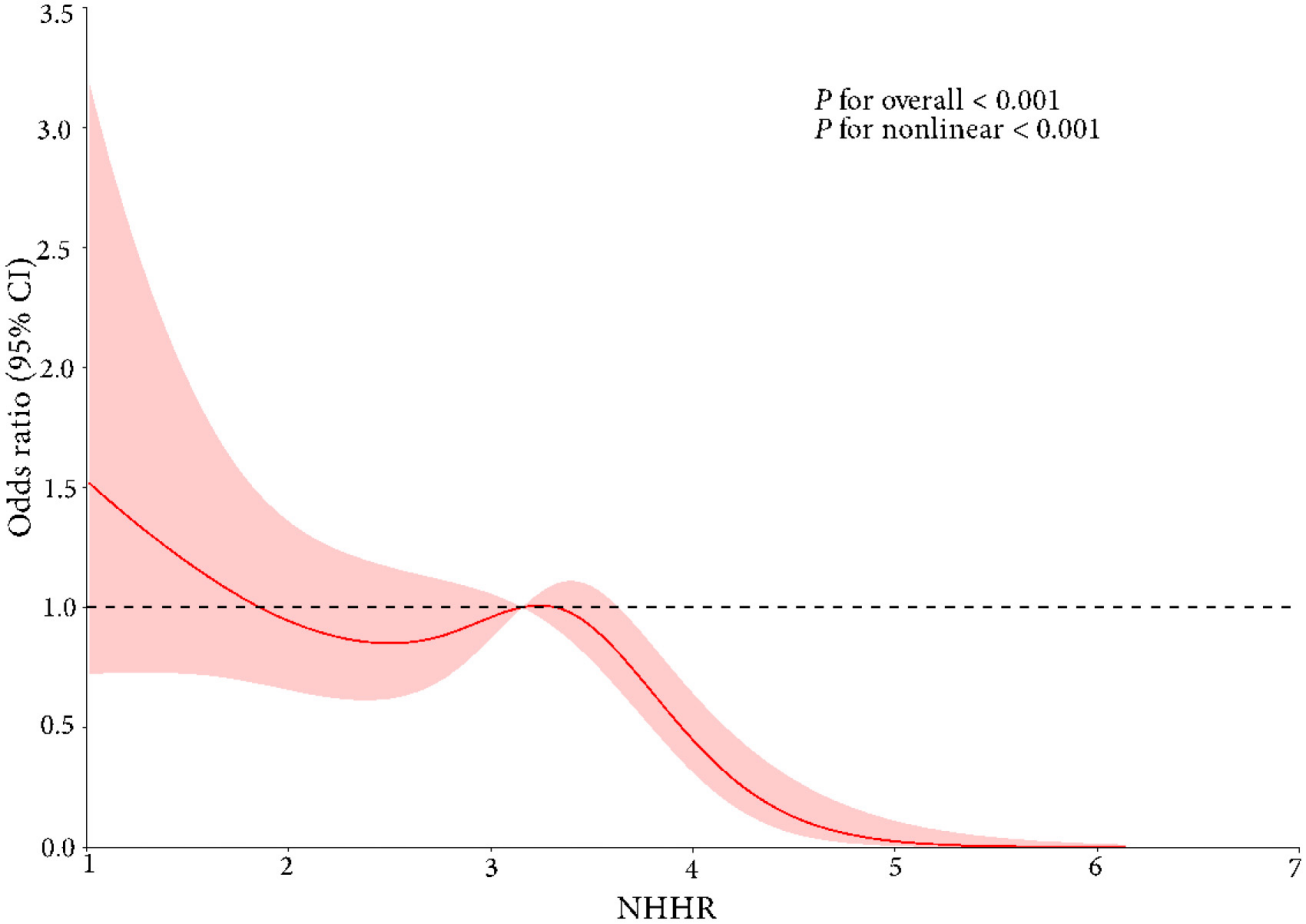
RCS curve fits the Association of NHHR with osteoporotic fracture. CI, confidence interval; NHHR, non-high-density lipoprotein cholesterol to high-density lipoprotein cholesterol ratio; OR, odds ratio. The solid red line represents the adjusted OR with the reference point set at the median NHHR value, modeled using RCS with four knots at the 5th, 35th, 65th, and 95th percentiles; The red shaded area indicates the 95% confidence interval of the OR estimates; The dashed horizontal line denotes the null effect (OR = 1); The overall association was statistically significant (*P* < 0.001), with evidence of non-linearity (*p* for non-linearity < 0.001). Covariates adjusted for: age, sex, BMI, hypertension, diabetes, OC, CTX, PTH, P, L1-L4 BMD, femoral neck BMD, AST, ALP, K, Na, Ca and Hb.

The dose-response relationship between the NHHR and risk of osteoporotic fracture was examined using RCS. RCS analysis with four knots placed at the 5th (1.51), 35th (2.74), 65th (3.38), and 95th (5.25) percentiles of NHHR distribution as indicated in Harrell's recommendations to balance model flexibility and overfitting risks (17). The results are shown in Figure 2 relatively higher risks of osteoporotic fractures were identified at lower NHHR levels, with a significant downward trend in fracture risk observed as NHHR increased. The OR values decreased markedly within the NHHR range of 3.38 to 5.25, suggesting a significantly lower risk of osteoporotic fracture at high NHHR levels. The relationship between NHHR and osteoporotic fracture risk was further explored using threshold effect analysis (Table 3). A non-linear relationship was identified with an inflection point at 3.29. The two-piecewise logistic regression analysis showed that when NHHR was below 3.29, there was no significant association with fracture risk (OR = 1.15, 95% CI: 0.79–1.68, *p* = 0.468). However, when NHHR exceeded 3.29, a significant protective effect was observed (OR = 0.13, 95% CI: 0.06–0.32, *p* < 0.001). The likelihood ratio test (*p* < 0.001) supported that the two-piecewise linear regression model was more appropriate than the standard linear mode.

**Table 3:** Applying a two-piecewise linear regression model to analyze the threshold effect of NHHR on osteoporotic fracture.

Model	OR (95% CI)	*p*-value
Inflection point (*K*)	3.29	
Fitting by two-piecewise linear model		
NHHR < 3.294	1.15 (0.79–1.68)	0.468
NHHR ≥3.294	0.13 (0.06–0.32)	< 0.001
*p* for likelihood test		< 0.001

### Subgroup analysis

**Figure 3:**
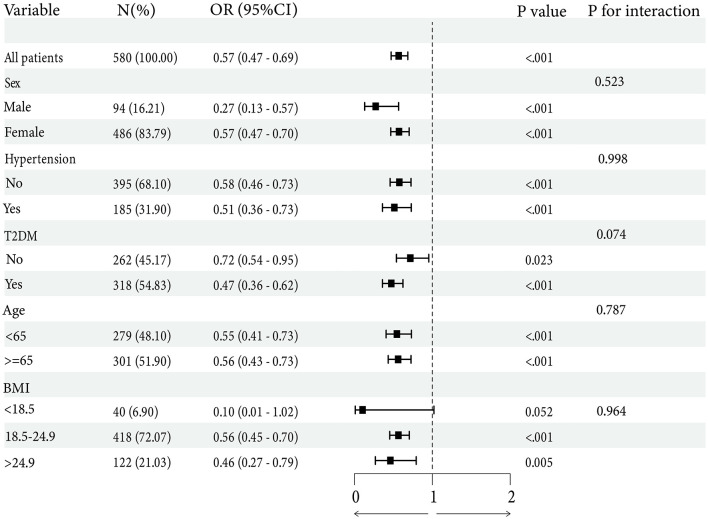
Subgroup analysis of the association between the NHHR and risk of Osteoporotic fracture. Subgroup analyses were stratified by age, sex, BMI, hypertension, and T2DM, with additional adjustment for covariates not included in each stratification.

To comprehensively assess the association between NHHR and osteoporotic fracture, subgroup analyses and interaction tests were conducted across sex, age, hypertension, T2DM, and BMI. The results of the subgroup analysis are presented in Figure 3. All analyses, except for the subgroup variable, were adjusted for these covariates. The findings showed that there were no statistically significant interaction across these subgroups (*p* for interaction >0.05). This consistency further supports the robustness of the observed protective association in the overall population.

## Discussion

In this study, we enrolled 580 participants to investigate the potential association between the NHHR and the likelihood of osteoporotic fractures. After adjusting for relevant covariates in continuous variable models, we identified a negative correlation between NHHR and osteoporotic fracture risk. This finding underscores the potential clinical significance of NHHR in the early identification of individuals at risk for osteoporotic fractures.

Currently, research examining the relationship between NHHR and osteoporotic fractures remains relatively scarce, although substantial evidence indicates a close association between lipid profiles and bone health. However, these research findings have not reached consensus, revealing complex regulatory relationships. The majority of studies demonstrate that HDL-C levels are negatively correlated with bone mineral density, with this association being particularly pronounced in women and individuals over 60 years of age. This phenomenon may be related to the direct influence of lipid metabolism on bone metabolism, though the specific mechanisms require further investigation. Deepening this understanding, a study on Chinese women revealed a non-linear relationship between serum TC, LDL-C, HDL-C and lumbar spine bone mineral density, with a negative correlation observed when these indicators fell below specific thresholds (18). This non-linear relationship suggests that lipids may exert a threshold effect on bone health. Concurrently, several studies indicate that lower LDL-C levels are associated with increased bone mineral density, and lipid-lowering medications (such as statins) may confer a protective effect on bone mineral density (19). However, these associations vary across different populations. Studies in healthy elderly populations have shown a significant correlation between elevated HDL-C levels and increased fracture risk (20). Conversely, a large-scale Korean study found a positive correlation between HDL-C and lumbar spine BMD in postmenopausal women (21). These conflicting findings suggest that the relationship between lipids and bone health may be modulated by multiple factors, including age, sex, and endocrine status. Recent technological advancements have brought new perspectives to this field. A nuclear magnetic resonance-based metabolomics study from the UK Biobank revealed that elevated levels of large and very large HDL particles are associated with an increased fracture risk, whereas LDL and VLDL subtype lipids correlate with reduced fracture risk and enhanced bone mineral density (22).

This subtyping research approach reveals subtle associations that traditional lipid analysis fails to capture. Synthesizing the aforementioned evidence, while conventional lipid indicators hold some reference value in assessing osteoporotic fracture risk, the relevant evidence remains contentious. Therefore, incorporating novel lipid parameters such as NHHR will provide a more comprehensive perspective for elucidating the complex relationship between lipids and osteoporotic fractures, potentially facilitating the development of more precise osteoporosis risk assessment tools and intervention strategies.

This study found that NHHR, after reaching a certain threshold, is associated with a decreased risk of osteoporotic fractures. From a biological perspective, this association may be related to the interplay between cholesterol metabolism, sex hormones, and bone remodeling. Firstly, an elevated NHHR indicates an increase in non-HDL-C or a decrease in HDL-C. On the one hand, elevated non-HDL-C levels may provide sufficient cholesterol substrate for the synthesis of sex hormones in the body (23). When cholesterol supply is adequate, the synthesis and secretion of sex hormones can be maintained at normal levels, which is crucial for bone metabolism (24). Specifically, estrogen promotes osteoblast differentiation and bone matrix production, primarily through the ERα-mediated Wnt/β-catenin signaling pathway and GPX4-regulated osteocyte redox homeostasis, thereby exerting its bone-protective effects (25–27). Furthermore, estrogen effectively maintains bone density by inhibiting osteoclast activity and attenuating bone resorption (28). Testosterone also plays a critical role in bone formation and the maintenance of bone tissue, primarily by directly enhancing osteoblast function. Moreover, its partial conversion to estrogen *in vivo* enables testosterone to synergistically contribute to bone-protective effects (29, 30). On the other hand, HDL-C plays a crucial role in peripheral cholesterol clearance and reverse cholesterol transport in the body. Studies have shown that HDL mediates the removal of cholesterol from peripheral tissues, promoting its return to the liver for metabolism (31). A decrease in HDL-C levels inhibits peripheral cholesterol clearance, resulting in the gradual accumulation of cholesterol in tissues such as bone (32), potentially supporting steroid hormone synthesis.

Taken together, these mechanisms provide a biologically plausible interpretative framework rather than a definitive causal explanation. Further mechanistic and prospective studies are needed to clarify this association.

Furthermore, our subgroup analysis revealed no significant association between NHHR and the risk of osteoporotic fracture in underweight individuals (*p* > 0.05), suggesting that body weight may have a moderating effect. Mechanical stimulation, as a critical physiological factor regulating bone metabolic balance and tissue homeostasis, is indispensable for maintaining bone health (33). Osteocytes can sense changes in mechanical loading and activate multiple signaling pathways, including Wnt/β-catenin and Piezo1, to promote bone formation or enhance bone resorption, thereby enabling the skeleton to dynamically adapt to external forces (34, 35). Relevant studies have shown that elderly individuals with lower body weight or a lean physique are at increased risk of fractures. In contrast, those with sufficient fat reserves and higher body weight tend to have a lower risk of fragility fractures, particularly in the hip. This protective effect is primarily attributed to increased mechanical loading associated with greater body weight, which stimulates bone remodeling and enhances bone density (36). In addition, individuals with higher traditional body composition indicators such as BMI generally exhibit greater bone density, whereas those with a leaner physique tend to have lower bone density, correspondingly increasing their risk of fractures (37). Recent studies suggest that elevated BMI may promote bone formation not only through increased mechanical loading, but also by modulating lipid metabolism, particularly via the mediating effects of HDL and VLDL, thereby affecting bone mineral density and fracture risk (38). Overall, body weight influences the relationship between NHHR and the risk of osteoporotic fractures not only through mechanical stimulation affecting bone metabolism but also potentially in synergy with lipid metabolic pathways. The specific regulatory mechanisms involved require further in-depth investigation.

### Strengths and limitations

Previous studies examining the relationship between lipid metabolism and osteoporotic fractures have primarily focused on individual lipid indicators. In contrast, this study utilized the comprehensive lipid parameter NHHR and identified a significant association with osteoporotic fracture risk. Rigorous adjustment for various potential confounders enhanced the reliability of the results. Additionally, subgroup analyses evaluated the consistency of this association across different populations, further strengthening the robustness of the conclusions.

Nonetheless, several limitations should be acknowledged. First, the cross-sectional design precludes the determination of temporal relationships between variables, thereby limiting causal inference and time-dependent risk evaluation. Second, the single-center and regional characteristics of the dataset may restrict the generalizability of the findings to other populations or ethnic groups. In particular, residual confounding related to medication use remains possible, as medications may influence both lipid profiles and bone metabolism. Future prospective cohort studies with longitudinal design, multi-center recruitment, balanced sampling, and comprehensive data collection are necessary to address these limitations.

## Conclusions

In conclusion, elevated NHHR levels are associated with a reduction in osteoporotic fracture risk, particularly when exceeding the inflection point of 3.29. As an easily obtainable lipid parameter, NHHR may serve as a valuable addition to current osteoporotic fracture risk assessment strategies, improving the identification of high-risk individuals and optimizing targeted preventive interventions. Further multicenter prospective studies are needed to validate these findings and clarify the underlying biological mechanisms.

## Data Availability

The raw data supporting the conclusions of this article will be made available by the authors, without undue reservation.
